# Telomere length in early childhood is associated with sex and ethnicity

**DOI:** 10.1038/s41598-019-46338-x

**Published:** 2019-07-17

**Authors:** Kien Ly, Caroline Walker, Sarah Berry, Russell Snell, Emma Marks, Zaneta Thayer, Polly Atatoa-Carr, Susan Morton

**Affiliations:** 1 Centre for Longitudinal Research - He Ara ki Mua and Growing Up in New Zealand, University of Auckland, Auckland, New Zealand; 2 School of Biological Sciences, Centre for Brain Research, University of Auckland, Auckland, New Zealand; 3 Department of Anthropology, Dartmouth College, Hanover, New Hampshire United States; 4 National Institute of Demographic and Economic Analysis, Faculty of Arts and Social Sciences, University of Waikato, Hamilton, New Zealand

**Keywords:** Quantitative trait, Epidemiology

## Abstract

Telomeres are repetitive DNA sequences at the end of chromosomes that function to protect chromosomes from degradation. Throughout the life course, telomere length decreases with age and is influenced by environmental factors and health conditions. This study aimed to determine the relative telomere lengths in a diverse cohort of about 4000 four-year-old children in New Zealand. Linear regression was used to investigate the relationship between telomere length, child gender, ethnicity, paternal age and deprivation. We observed substantial variation in telomere length according to sex and self-identified ethnicity. Telomere length was longer in females compared to males (coefficient of 0.042, 95% confidence interval (CI) 0.024–0.060). European children had shorter telomere than both the indigenous Māori (coefficient of 0.03, CI 0.007–0.055) and Pacific children (coefficient of 0.15, CI 0.12–0.18). The data suggest that telomere lengths are highly variable and variability between individuals arise from early age, influenced partly by sex and ethnicity. Longer telomeres in indigenous Māori and Pacific children may reflect the heritability of telomere length in genetically less complex populations. This study increases our understanding of telomere dynamics in young children since the majority of telomere studies are conducted in adults.

## Introduction

Telomeres are regions of repetitive DNA composed of the conserved sequence of TTAGGG and associated proteins located at chromosomal ends^1^. The DNA and proteins form a loop structure and serve to protect the ends of chromosomes from end-to-end fusion, recombination and degradation^2^. Due to the inability of DNA polymerase to completely replicate the ends of chromosomal DNA, termed the “end-replication-problem”^3^, telomeres get shorter at each cycle of cell division. Telomere length has therefore been found to be negatively associated with chronological age^4–6^. The rate of telomere length shortening can be influenced by a myriad of additional factors. For example, telomere length has been found to be negatively associated with obesity^7,8^, diabetes^9^, some cancers^10^, and cardiovascular disease^11^. Additionally, telomere length is negatively correlated with mental health factors such as perceived stress^12,13^ and loneliness, as well as behaviours and external environmental conditions, such as exposure to violence^14^, smoking^15^, socioeconomic status^16^, and social interaction^17^. Sex differences have also been observed in telomere length with females having longer telomeres compared to males^18,19^. Furthermore, telomere length appears to be a heritable trait, contributed to by several genetic loci^20,21^ as well as epigenetic regulation^22^.

Even though there have been a large number of studies looking at telomere length in adult populations, studies on telomere length in children, with a view to tracking telomere length throughout development and adulthood are comparatively few. Our objective was to measure telomere length in children from the *Growing Up in New Zealand* longitudinal cohort study^23^. Further, we aimed to determine the baseline telomere length patterns in this longitudinal cohort so that future correlations with health and wellbeing could be ascertained. Telomere lengths can be measured by several methodologies^24^: terminal restriction fragment, quantitative fluorescence *in Situ* hybridization, quantitative polymerase chain reaction (qPCR), each with their own strengths and weaknesses. In this study, relative telomere lengths were determined using a qPCR-based method^25^ by comparing the telomere signal to that of a single copy gene (T/S ratio).

## Results

In this cohort, 6156 participants took part in the 4 year data collection wave. In total, 4587 (75%) saliva samples were collected using the DNAgenotek Oragene 575 kit. From these, DNA from 4384 samples (96% of samples) was successfully extracted and measured for telomere length measurement (Table 1). Across the whole cohort, the mean natural log transformed T/S ratio was 0.306 (±0.0047 SE) with a minimum of −1.009 and a maximum of 1.604.

**Table 1:** Log transformed relative telomere length across participants who consented and provided a saliva sample, partitioned by child ethnicity and sex.

Ethnicity	Sex	Number of samples	Mean	Standard error
All	Male	2296	0.285	0.0067
	Female	2088	0.328	0.0065
European	Male	984	0.251	0.0099
	Female	909	0.289	0.0099
Māori	Male	587	0.275	0.0135
	Female	525	0.316	0.0127
Pacific People	Male	320	0.403	0.0177
	Female	296	0.431	0.0167
Asian	Male	300	0.307	0.0173
	Female	268	0.362	0.0176
Other	Male	105	0.242	0.0325
	Female	90	0.353	0.0301

**Figure 1:**
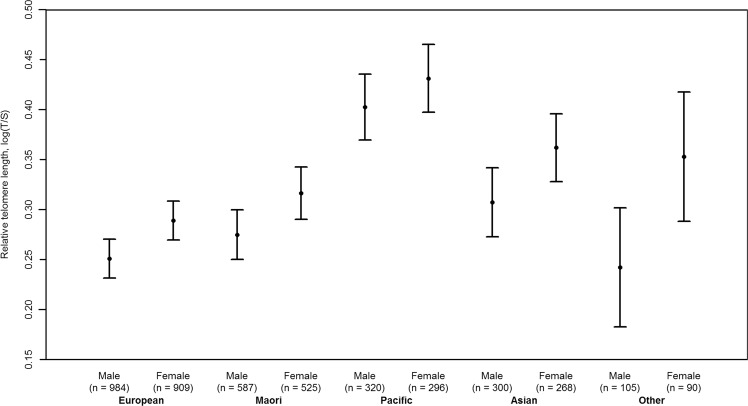
Relative telomere length expressed as log transformed T/S ratio across sex and ethnicity (mean and 95% CI).

Univariate analysis revealed that relative telomere lengths were associated with sex, ethnicity, maternal age, paternal age and area level socioeconomic deprivation (Table 2). The relative telomere lengths of girls were higher than those of boys (p-value of 3.75 × 10^−06^, effect size of 0.0429). Europeans had the shortest relative telomere length while Pacific children possessed the longest (p-value of 2.74 × 10^−25^) (Fig. 1). Homoscedasticity across samples among the ethnic groups were tested (p-value of 0.7369, Bartlett test of homogeneity of variances; p-value of 0.881, Levene test). Increased parental age, both maternal and paternal, was correlated with longer relative telomere length, with a p-value of 1.27 × 10^−02^ and 1.52 × 10^−05^ respectively. The effect of area level socioeconomic deprivation was also statistically significant in the univariate analyses with those living in the most deprived areas having a longer telomere length compared to those living in the least deprived areas (p-value of 6.12 × 10^−05^).

**Table 2:** Univariate analysis of selected variables predicting log(T/S).

Variable	Level	Coefficient	Standard error	p-value
Sex	Male (ref)	0.2853	0.0064	<2e-16
	Female	0.0429	0.0093	3.75e-06
Child ethnicity	European (ref)	0.2693	0.0070	<2e-16
	Māori	0.0252	0.0115	0.0281
	Pacific People	0.1471	0.0141	2.74e-25
	Asian	0.0638	0.0145	1.13e-05
	Other	0.0240	0.0228	0.292
Maternal age	(Intercept)	0.2460	0.0245	<2e-16
		0.0020	0.0008	0.0127
Paternal age	(Intercept)	0.1668	0.0297	<2e-16
		0.0038	0.0009	1.52e-05
Deprivation	Low (ref)	0.2905	0.0084	<2e-16
	Medium	−0.0019	0.0114	0.864
	High	0.0468	0.0117	6.12e-05

**Figure 2:**
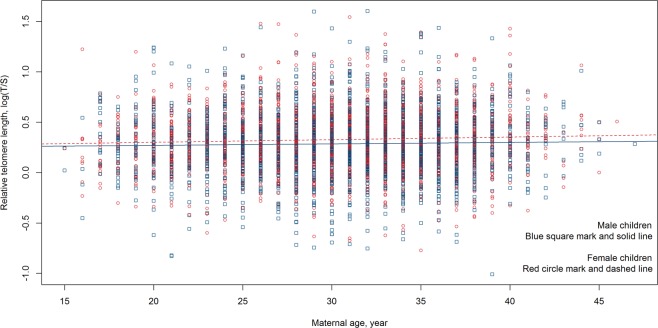
Relative telomere length expressed as log transformed T/S ratio versus maternal age. Male children: blue square mark and solid line; female children: red circle mark and dashed line.

Given maternal and paternal age were highly correlated (Pearson coefficient of 0.714) and that we have many more observations for maternal age compared to paternal age, we excluded paternal age from subsequent multivariable analyses. Child sex, ethnicity, maternal age and area level socioeconomic deprivation was regressed against T/S ratio in a multivariable analysis which resulted in a model where sex, ethnicity and maternal age had independent effects (Table 3). In the multivariable model relative telomere lengths of female children were on average 4% longer than those of male children (p-value of 5.22 × 10^−06^). For each year increase in maternal age, relative telomere lengths in the children increased by 0.4% (Fig. 2). Māori, Pacific and Asian children all exhibited longer relative telomere length compared to European children, with p-values of 0.011, 3.77 × 10^−22^, and 2.91 × 10^−06^, respectively. On average, telomere lengths of Māori, Pacific and Asian children were 3%, 16% and 7% longer than European children, respectively. Children grouped in the ‘Other’ ethnicity category had telomere lengths comparable to that of European children. Because the ‘Other’ group is highly heterogeneous, comprising Middle Eastern, Latin American, African, we thus assessed the model again excluding this group (N = 195). Removal of this group of children from the dataset did not change the observed differences detected between genders, ethnicities or maternal age (Table 4). Area level socioeconomic deprivation became non-significant when the other variables (sex, ethnicity and maternal age) were taken into account. Thus in the final model, three variables, sex, ethnicity and maternal age, had independent effects on relative telomere length.

**Table 3:** Multivariable analysis with the model: log(T/S) ~sex + ethnicity + maternal age + deprivation.

Variable	Level	Coefficient	Standard error	p-value
(Intercept)		0.1211	0.0294	3.86e-05
Sex (ref: Male)	Female	0.0420	0.0092	5.22e-06
Ethnicity (ref: European)	Māori	0.0313	0.0123	0.011
	Pacific People	0.1505	0.0155	3.77e-22
	Asian	0.0688	0.0147	2.91e-06
	Other	0.0252	0.0230	0.272
Maternal age		0.0040	0.0008	1.71e-06
Deprivation (ref: low)	Medium	−0.0051	0.0114	0.654
	High	0.0191	0.0130	0.142

All 5 categories of ethnicities (European, Māori Pacific People, Asian and Other) were included in the analysis.

**Table 4:** Multivariable analysis with the model: log(T/S) ~sex + ethnicity + maternal age + deprivation.

Variable	Level	Coefficient	Standard error	p-value
(Intercept)		0.1217	0.0299	4.81e-05
Sex (ref: Male)	Female	0.0390	0.0094	3.46e-05
Ethnicity (ref: European)	Māori	0.0304	0.0123	0.0139
	Pacific People	0.1493	0.0155	9.96e-22
	Asian	0.0681	0.0147	3.59e-06
Maternal age		0.0040	0.0008	3.19e-06
Deprivation (ref: low)	Medium	−0.0014	0.0118	0.9073
	High	0.0223	0.0133	0.0926

The “Other” level in the ethnicity variable was excluded so only the remaining 4 categories of ethnicities (European, Māori Pacific People, Asian) were used in the analysis.

## Discussion

In this study, we have measured relative telomere length in a diverse cohort of children born in New Zealand. To date, this is the largest study of telomere lengths in New Zealand and also the first study of telomere length in preschool children in New Zealand. The data revealed substantial variation in relative telomere lengths among the participants at 4 years of age. This level of variation appears to be consistent with other studies which similarly reported a large degree of variation in telomere length^12,17,20,21,26^.

We found that female children possessed longer relative telomere lengths than male children at four years of age, and this remained significant after adjusting for ethnicity and maternal age. This is consistent with a previous review that reported females have longer telomere lengths compared to age matched males^18^. The present research is the first to report sex differences in telomere length in young children in New Zealand. Previous studies (sample sizes ranging from 160 to 800) investigating sex differences in telomere length have not consistently reported a significant difference between sexes in children^7,27–29^. Sample size and measurement technique are key differences in studies reporting inconsistent results in regards to sex. A recent meta-analysis reported that studies where telomere length was assessed by Southern blot, are more powerful in detecting telomere length variations than the qPCR based method^18^. The ability to detect sex-specific difference in telomere length in our study, which also utilizes qPCR, is likely due to the larger sample size in the *Growing Up in New Zealand* cohort. Our study suggests that differences in telomere length between genders arise at an early age. The differences are statistically significant but the effect size is relatively small compared to the differences reported for older adults. Future studies of this cohort as they age will enable us to determine if telomere length differences between genders get larger across the life course.

Maternal age was associated with relative telomere length in 4 year old children, such that children with older mothers had longer telomeres. This is consistent with earlier studies reporting positive correlations between parental age and offspring’s telomere length^30–32^. We report a similar effect size for maternal age as a recent meta-analysis encompassing nearly 20 thousands subjects^33^. Our results are consistent with the established literature and demonstrate that the positive association between maternal age and offspring telomere length is present and has a similar effect size from an early age.

Telomere length differed between children by parental ascribed ethnic identification. On average, those children identified as Pacific had the longest telomere length, followed by Asian and Māori children. European children and those in the ‘Other’ ethnic group had similar telomere lengths and both were, in general, shorter than Māori, Pacific and Asian children. Three independent studies of 1500 to 5000 participants using either qPCR based approach or Southern blots consistently found that Caucasian Americans have shorter telomere lengths compared to African Americans after adjusting for covariates such as age, sex, and BMI^34–36^. A study of approximately 800 individuals from 11 European countries using the qPCR based method also showed substantial telomere length variation in different ethnic populations after controlling for age^26^. A recent study using a similar methodology on a New Zealand cohort of about 700 adult participants also found that Māori and Pacific populations had significantly higher mean relative telomere lengths compared to European people^37^. Therefore, the difference in telomere length by ethnicity observed in our study is in alignment with other contemporary reports. Differences in telomere length between ethnicities may be the result of genetic variation. Further research investigating the interaction between genetic and environmental factors that may mediate the association between telomere length and ethnicity is therefore warranted^20–22^. In addition, it is important to note that ethnicity assignment in our study is self-prioritized as reported by the primary caregivers when the children were a 4 years old. Ethnicity is a social construct not a biological construct in the New Zealand context.

To our knowledge, this is the first study investigating relative telomere length in early childhood in New Zealand. Secondly, the study shows that telomere length difference between sexes exists for children in New Zealand at this age. Thirdly, this is the most comprehensive assessment of telomere length in Māori, Asian and Pacific children in New Zealand to date, showing differences in telomere length between ethnic groups independent of maternal age, area level socioeconomic deprivation exposure, and sex. These are significant findings as we were able to replicate sex specific difference in telomere length in young children as well as ethnic specific differences in the adult population. We also discovered new telomere length variation in children. Future studies using the unique and diverse cohort of children from *Growing Up in New Zealand* will enable longitudinal studies of how telomere length changes over time between sexes, ethnicity and how this is associated with health and longevity. Māori and Pacific people suffer from lower life expectancy and higher disease burden compared to European in New Zealand^38^, yet Māori and Pacific children have longer relative telomere length compared to European children. Further research is needed to better understand the role of telomere length and its interaction with other potential genetic and environmental factors in health and wellbeing among Māori and Pacific peoples.

## Material and Methods

### Cohort

A profile of the cohort has been described previously in detail^23^. Essential design features of *Growing Up in New Zealand* include antenatal recruitment, inclusion of partners and engagement with a diverse population, particularly with respect to ethnic identification and socioeconomic characteristics. Invitations to participate were made to all eligible pregnant women with an expected delivery date between April 2009 and March 2010 who were living in three of the District Health Board regions of New Zealand (Auckland, Counties-Manukau and Waikato) using a variety of strategic and monitored recruitment strategies^39^. There were no other inclusion or exclusion criteria. These regions were selected as their demographics closely represented the diversity of contemporary New Zealand births without the requirement for over sampling or weighting^39^. The enrolled cohort has been shown to represent 1/3 births within the study region, and 11% of births within New Zealand during the study period, and the resulting main child cohort are generally comparable to New Zealand national birth statistics, especially in relation to key parameters such as maternal age and parity, maternal ethnicity, and area-level deprivation^40^. The recruitment of 6822 women led to a birth cohort of 6853 children. Ethical approval for the study was obtained from the Ministry of Health Northern Y Regional Ethics Committee. Written informed consent was obtained from parents of the participants in the study. This study was conducted in accordance with the relevant guidelines and regulations.

### Sample

The sample reported here consisted of 4572 of the 6853 children participating in *Growing Up in New Zealand*. These children were those who had complete ethnic, social and environmental data since before birth and throughout the first 4 years of life as well as a saliva sample for DNA extraction. Total child ethnic identification was described by their mothers at the 4 year data collection wave, with children then categorised as European, Māori, Pacific, Asian or Other (this category included those children identified as Middle Eastern, Latin American and African (MELAA) and New Zealander). When more than one ethnicity was given, prioritised ethnicities were assigned with the following order: Māori > Pacific People > Asian > Other > European. Socio-economic deprivation was determined based on an area level measure combining census data relating to income, home ownership, employment, qualifications, family structure, housing, access to transport and communications; and providing a deprivation score for each small geographic area in New Zealand (NZDep2013). Decile 1 of NZDep2013 represents the areas with the least deprived scores, and decile 10 the most deprived 10% of areas in New Zealand^41^. Here we have classified the socioeconomic deciles as low (NZDep 1–3), medium (NZDep 4–7) and high (NZDep 8–10).

### Sample collection

Saliva samples for DNA were collected when the participants were 4 years old. Saliva samples were collected by trained interviewers at the participant’s home using the Oragene DNA/saliva Self-Collection kit (OG-575, DNA GenoTek Inc.) and DNA extraction was performed based on the manufacturer’s protocol. Extracted DNA were re-suspended in TE (10 mM Tris, 1 mM EDTA, pH 8.0) and stored at −80 °C. The quality of the extracted DNA was assessed using a photo-spectrometer and gel electrophoresis.

### Measurement of relative telomere length

Relative telomere length was determined by qPCR based on a previously published protocol^25^. Each reaction consisted of 7.5 μL of a 2× master mix (SYBR® FAST qPCR Kits, Kapa Biosystems), 0.5 μM of each of the primers (telg, telc, albu, albd) and ~10 ng of purified genomic DNA to yield a final reaction volume of 15 μL. Reagent and sample dispensing were carried out by a semi-automated liquid handling system (epMotion 5073, Eppendorf). Reactions were performed in 384-well plates. Each plate contained a 6-point standard curve from 0.05 ng to 10 ng of genomic DNA. The thermal cycling conditions were 1 cycle at 95 °C for 20 seconds, 40 cycles of 95 °C for 1 second and 56 °C for 20 seconds and 72 °C for 1 second with signal acquisition, and 88 °C for 1 second with signal acquisition, performed in a Roche LightCycler 480 II. Each sample was measured in triplicate. The primer sequences (5′ → 3′) for telomere amplification were telg (G rich) (ACACTAAGGTTTGGGTTTGGGTTTGGGTTTGGGTTAGTGT) and telc (C rich) (TGTTAGGTATCCCTATCCCTATCCCTATCCCTATCCCTAACA); for amplification of the single copy reference gene albumin were albu (albumin upstream) (CGGCGGCGGGCGGCGCGGGCTGGGCGGAAATGCTGCACAGAATCCT) and albd (albumin downstream) (GCCCGGCCCGCCGCGCCCGTCCCGCCGGAAAAGCATGGTCGCCTGTT).

Threshold cycle values were calculated from the raw data produced by LightCycler 480 II using the R package qpcR^42^. The package performs sigmoidal model selection from real time qPCR data analysis to produce C_t_ values. The default parameter using the function modlist were used for C_t_ calculation. Samples with a coefficient of variation higher than 2.5% were repeated. The overall coefficient of variation for the telomere sequence was 0.98%, for the reference albumin gene was 0.78%. The average amplification efficiency and coefficient of determination (R^2^) for the telomere sequence was 99.87% and 0.9993, and for the reference gene was 99.40% and 0.9992. The relative telomere length for each sample was expressed in T/S (telomere over single copy number gene) ratio and was calculated using the −∆∆C_t_ formula with efficiency taken into account: ${\rm{T}}/{\rm{S}}=\frac{{{{\rm{E}}}_{{\rm{tel}}}}^{({{\rm{C}}}_{{\rm{t}}}{\rm{tel}}-{\rm{standard}}-{{\rm{C}}}_{{\rm{t}}}{\rm{tel}}-{\rm{sample}})}}{{{{\rm{E}}}_{{\rm{alb}}}}^{({{\rm{C}}}_{{\rm{t}}}{\rm{alb}}-{\rm{standard}}-{{\rm{C}}}_{{\rm{t}}}{\rm{alb}}-{\rm{sample}})}}$ where E_tel_ and E_alb_ are the amplification efficiency of the telomere and albumin sequence respectively, C_t_tel-standard and C_t_tel-sample are the C_t_ for the telomere sequence of the standard DNA and that of the sample respectively, C_t_alb-standard and C_t_alb-sample are the C_t_ for the telomere sequence of the standard DNA and that of the sample respectively. The T/S ratio is expected to be proportional to the average telomere length per cell. A T/S ratio >1 indicates that the measured sample has an average telomere length greater than that of standard DNA. Conversely, a T/S ratio <1 indicates that the sample has an average telomere length shorter than that of standard DNA. T/S ratios were natural log transformed to achieve normality before we undertook further analyses.

### Statistical analysis

Linear regression analyses were performed using R and its base packages (version 3.4)^43^. T/S ratio was used as the dependent variable in univariate analysis using the following variables: sex, ethnicity, parental ages (maternal and paternal) and area-level deprivation. The significance level (alpha) was set at 0.05. Variables reaching statistical significance were included in subsequent multivariable analyses. Variables were removed from multivariate analyses when they dropped below the significance level.

## Data Availability

The datasets generated during and/or analysed during the current study are available through the application to the Growing Up in New Zealand Data Access Committee.
